# The complete chloroplast genome of *Sonneratia griffithii* Kurz (Lythraceae)

**DOI:** 10.1080/23802359.2022.2119818

**Published:** 2022-10-03

**Authors:** Duangjai Sangsrakru, Chutima Sonthirod, Wanapinun Nawae, Chutintorn Yundaeng, Waratthaya Promchoo, Wirulda Pootakham, Sithichoke Tangphatsornruang

**Affiliations:** a National Omics Center, National Science and Technology Development Agency (NSTDA), Pathum Thani, Thailand; b Department of Marine and Coastal Resources, Royal Thai Government Ministry of Natural Resources and Environment, Bangkok, Thailand

**Keywords:** Chloroplast genome, phylogeny, mangrove, *Sonneratia griffithii*

## Abstract

*Sonneratia griffithii* Kurz is a critically endangered mangrove species that can be found along the western coast of Thailand. In this study, we reported the complete chloroplast genome of *S. griffithii*. The chloroplast genome is 152,730 bp, consisting of one large single-copy (LSC) region, one small single-copy (SSC) region and a pair of inverted repeats (IRs). The LSC, SSC, and IR lengths are 87,226, 17,764, and 23,870 bp, respectively. The genome contains 113 unique genes, including 79 protein-coding, 30 tRNA, and 4 rRNA genes. The GC content of the chloroplast genome is 37.31%. The phylogenetic analysis based on 76 protein-coding genes showed a monophyletic group of *S. griffithii* and other *Sonneratia* species.

*Sonneratia griffithii* (Kurz 1871) is a true mangrove in the family Lythraceae. It is categorized as a rare and critically endangered species under the International Union for Conservation of Nature (IUCN) (Duke et al. 2010). *Sonneratia griffithii* can be found along the coasts of Bengal and the Andaman Sea in India, Myanmar, Malaysia, and Thailand (Kathiresan and Rajendran 2005). In Thailand, *S. griffithii* is distributed near the western coast in Ranong, Phang-nga, Krabi, and Trang provinces. *Sonneratia* variations have been reported by a natural hybridization between *S. griffithii* and *Sonneratia alba* (Qiu et al. 2008). Understanding genetic diversity is important for *Sonneratia* conservation and for clarifying the evolution of this mangrove species. In this paper, we report the complete chloroplast genome of *S. griffithii*, which provide a useful resource for genetic diversity studies. We also performed a phylogenetic analysis to demonstrate the relationships between *S. griffithii* and other mangrove species.

*Sonneratia griffithii* leaves were collected from a mature plant in the Ranong Mangrove Forest Research Center, Ranong Province, Thailand (10°10′20.3″N, 98°42′31.4″E), following the guidelines on the implementation of the ‘IUCN Policy Statement on Research Involving Species at Risk of Extinction’ (June 1989). Sample collection for this study was permitted by the Department of Marine and Coastal Resources, Ministry of Natural Resources and Environment, Thailand (project number 1952261). The analysis of chloroplast DNA was followed protocols in Ruang-Areerate et al. (2021). Leaf tissues were frozen in liquid nitrogen after being collected and genomic DNA was extracted from young leaves using the MagAttract HMW DNA Kit (Qiagen, Germany). The DNA sample was deposited in the National Biobank of Thailand (NBT), Thailand Science Park, Pathum Thani, Thailand (contact person: Panyavut Aumpuchin; Email: panyavut.aum@nstda.or.th) under the voucher number NBTG000002. Paired-end (PE) reads of 150 bp were conducted on an Illumina HiSeq X Ten platform (Illumina, USA). After quality assessment, the 101,166,742 raw reads were used to assemble the chloroplast genome using GetOrganelle v1.7.3.5 (Jin et al. 2020), and the assembly was annotated with GeSeq (Tillich et al. 2017). The complete chloroplast genome sequence of *S. griffithii* was submitted to the GenBank database with accession number OL628854.

The complete chloroplast genome of *S. griffithii* contained 152,730 nucleotides with a GC content of 37.31%. The genome had a large single copy (LSC) region with a length of 87,226 bp and a small single copy (SSC) region of 17,764 bp. These single-copy regions were separated by a pair of 23,870-bp inverted repeats (IRs). In total, 113 unique genes were predicted, including 79 protein-coding genes, 30 tRNA genes, and 4 rRNA genes. There were 16 genes (*atpF*, *ndhA*, *ndhB*, *petB*, *petD*, *rpl16*, *rpoC1*, *rps12*, *rps16*, *rrn23*, *trnA-UGC*, *trnG-UCC*, *trnI-GAU*, *trnK-UUU*, *trnL-UAA,* and *trnV-UAC*) containing one intron and 2 genes (*clpP1* and *pafI*) having two introns.

A phylogenetic tree was constructed from 15 species in the family Lythraceae (including 5 *Sonneratia* species and 3 inter-specific hybrids) and 8 other mangrove species. *Vistis vinifera* was used as an outgroup species. The complete chloroplast sequences were downloaded from the NCBI (www.ncbi.nlm.nih.gov). A total of 76 conserved orthologs were identified, and the maximum-likelihood phylogenetic tree was constructed using RAxML v8.2.12 (Stamatakis 2014). The bootstrap support values in the phylogenetic tree were between 83 and 100% (except for the *Sonneratia* group), suggesting a confident species grouping in the tree. Based on our phylogenetic tree, *S. griffithii* was closely related to *S. alba* and was placed in a monophyletic group with *Sonneratia* species, while *Trapa* species were placed in a sister group (Figure 1). The data reported in this study are useful for genetic conservation as well as for phylogenetic studies of mangrove species.

**Figure 1.**
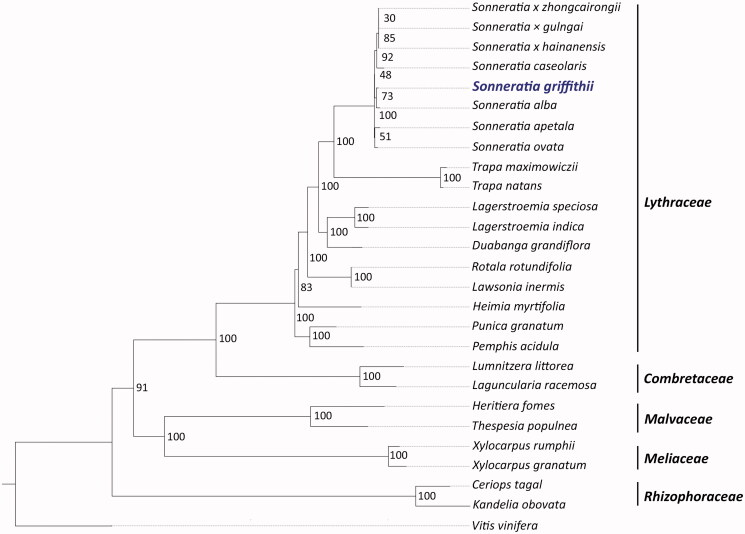
A maximum-likelihood phylogenetic tree based on 76 orthologs from the chloroplast genomes. *V. vinifera* was an outgroup. Numbers on the nodes represent bootstrap values. The following sequences were used: *Sonneratia × gulngai* NC_062075, *Sonneratia × zhongcairongii* NC_062167, *Sonneratia × hainanensis* NC_062073, *Sonneratia caseolaris* MN990684, *Sonneratia griffithii* OL628854, *Sonneratia alba* NC_039975 (Yan et al. 2019), *Sonneratia apetala* MH986669 (Wang and Ren 2022), *Sonneratia ovata* MW266118 (Wang and Ren 2022), *Trapa natans* NC_042895 (Fan et al. 2022), *Trapa maximowiczii* NC_037023 (Xue et al. 2017), *Lagerstroemia indica* NC_030484, *Lagerstroemia speciosa* NC_031414 (Gu et al. 2016), *Duabanga grandiflora* NC_042899, *Lawsonia inermis* NC_042369, *Rotala rotundifolia* NC_042888, *Heimia myrtifolia* MG921615 (Gu et al. 2018), *Punica granatum* NC_035240 (Rabah et al. 2017), *Pemphis acidula* NC_041439 (Jian and Ren 2019), *Lumnitzera littorea* NC_039752 (Zhou et al. 2018), *Laguncularia racemosa* NC_042719, *Heritiera fomes* NC_043924, *Thespesia populnea* NC_048518, *Xylocarpus rumphii* NC_038199, *Xylocarpus granatum* NC_039925, *Ceriops tagal* OK258322 (Ruang-areerate et al. 2022), *Kandelia obovata* NC_042718, *Vitis vinifera* NC_007957 (Jansen et al. 2006).

## Author contributions

DS, WP^a^ and ST designed research study and obtained the funding. DS, CY and WP^b^ performed laboratory work (sample collection, DNA extraction, library construction and sequencing). CS and WN performed bioinformatics analyses. DS wrote and revised the manuscript, and all authors reviewed it.

## Data Availability

The data that support the findings of this study are openly available in the GenBank database https://www.ncbi.nlm.nih.gov/genbank/under the accession number OL628854. The associated BioProject, SRA, and Bio-Sample numbers are: PRJNA783371, SRR17035265, and SAMN23429313 respectively.
